# The effect of balance training on postural control in people with multiple sclerosis using the CAREN virtual reality system: a pilot randomized controlled trial

**DOI:** 10.1186/s12984-016-0124-y

**Published:** 2016-03-01

**Authors:** Alon Kalron, Ilia Fonkatz, Lior Frid, Hani Baransi, Anat Achiron

**Affiliations:** Department of Physical Therapy, School of Health Professions, Sackler Faculty of Medicine, Tel-Aviv University, Tel-Aviv, Israel; The Multiple Sclerosis Center, Sheba Medical Center, Tel-Hashomer, Israel; The Center of Advanced Technologies in Rehabilitation, Sheba Medical Center, Tel-Hashomer, Israel; Sackler Faculty of Medicine, Tel-Aviv University, Tel-Aviv, Israel

**Keywords:** Multiple sclerosis, Balance, Postural control, Virtual reality, CAREN

## Abstract

**Background:**

Multiple sclerosis (MS) is a multi-focal progressive disorder of the central nervous system often resulting in diverse clinical manifestations. Imbalance appears in most people with multiple sclerosis (PwMS). A popular balance training tool is virtual reality (VR) with several advantages including increased compliance and user satisfaction. Therefore, the aim of this pilot RCT (Trial registration number, date: ISRCTN14425615, 21/01/2016) was to examine the efficacy of a 6-week VR balance training program using the computer assisted rehabilitation environment (CAREN) system (Motek Medical BV, Amsterdam, Netherlands) on balance measures in PwMS. Results were compared with those of a conventional balance exercise group. Secondary aims included the impact of this program on the fear of falling.

**Methods:**

Thirty-two PwMS were equally randomized into the VR intervention group or the control group. Each group received balance training sessions for 6 consecutive weeks, two sessions per week, 30 min sessions. Clinical balance tests and instrumented posturography outcome measures were collected upon initiation of the intervention programs and at termination.

**Results:**

Final analysis included 30 patients (19 females, 11 males; mean age, (S.D.) = 45.2 (11.6) years; mean EDSS (S.D.) = 4.1 (1.3), mean disease duration (S.D.) = 11.0 (8.9) years). Both groups showed a main effect of time on the center of pressure (CoP) path length with eyes open (F = 5.278, *P* = .024), sway rate with eyes open (F = 5.852, *P* = .035), Functional Reach Test (F = 20.841, *P* = .001), Four Square Step Test (F = 9.011, *P* = .031) and the Fear of Falls self-reported questionnaire (F = 17.815, *P* = .023). In addition, significant differences in favor of the VR program were observed for the group x time interactions of the Functional Reach Test (F = 10.173, *P* = .009) and fear of falling (F = 6.710, *P* = .021).

**Conclusions:**

We demonstrated that balance training based on the CAREN device is an effective method of balance training for PwMS.

## Background

Imbalance is present and evident in most people with multiple sclerosis (PwMS) and can appear as the initial symptom of MS [1], even in those with minimal changes on clinical examination [2, 3]. In most cases, as the disease progresses, balance difficulties persist and become more pronounced [4, 5]. Moreover, poor balance control is known as one of the main risk factors for falls and an elevated fear of falling [6, 7].

Intervention programs directed at improving balance control have employed various approaches, e.g. motor and sensory strategies [8], Feldenkrais exercises [9] and neuromuscular facilitation [10]. According to a recent systematic review, balance may improve in PwMS through exercise interventions [11]. However, the authors note that disparities in interventions, outcomes and methodologies imply that the results must be viewed with caution. Thus, given the adverse consequences of impaired balance, additional intervention strategies to reduce this phenomenon are still warranted.

In this situation, balance training together with a virtual reality (VR) system can provide training in a stimulating and enriching environment [12]. VR can supply immediate feedback as to performance, thus assisting with the learning of new motor strategies of movement and is an acceptable approximation of the real world (e.g. walking on an uneven or slippery surface, walking in a crowded area, etc.) [13]. Additionally, due to the diverse settings and game situations placed in many VR systems, compliance, retention and user satisfaction are increased and are therefore, potentially beneficial for long-term effectiveness of rehabilitation programs [14].

VR balance training has been shown to improve balance capabilities in the elderly, in patients following stroke and people with Parkinson’s disease [15–17]. According to a recent systematic review investigating the efficacy of VR training in stroke survivors, dynamic balance improved significantly following VR-based interventions as compared to other interventions [18]. Furthermore, VR-based interventions favorably affected participants in terms of dealing with environmental challenges, which may also facilitate independent community ambulation [18].

Justifiably, VR balance training could be useful for PwMS. Yet, only a few reports have investigated this intervention tool in the MS population [19–23]. Although results of these reports are promising, all favoring the efficacy of VR balance training; most studies were relatively small and without a comparison group.

In the present randomized controlled trial (RCT), we examined the effects of balance training utilizing the computer assisted rehabilitation environment (CAREN) system (Motek Medical BV, Amsterdam, Netherlands). Recently, balance training, using the CAREN system, showed an improvement in balance performance in persons with traumatic brain injury [24] and individuals with transtibial amputation [25].

To date, no RCT publication examining this system in the MS community was found in the Pubmed database. Therefore, the aim of this RCT was to examine the efficacy on balance measures in PwMS for a 6-week VR balance training program using the CAREN system. Results were compared with those of a conventional balance exercise group. Secondary aims included the impact of this program on the fear of falling. We hypothesized that following the intervention period, both groups would demonstrate improved balance capabilities; nonetheless, we expected greater improvements in the VR training group.

## Methods

### Participants

The implemented study design was executed according to the rigor of the CONSORT guidelines [26]. The RCT was a pilot, prospective, assessor blinded, parallel group, performed at the Multiple Sclerosis Center, Sheba Medical Center, Tel-Hashomer, Israel, between June 2014 and May 2015. Eligible PwMS were enrolled according to the following criteria: (1) diagnosis of definite relapsing-remitting MS according to the revised McDonald criteria 2010 [27], (2) 25–55 years of age, (3) moderate neurological disability as scored by the expanded disability status scale (EDSS), ranging from 3.0 to 6.0 inclusive with a pyramidal functional score of at least 3. Exclusion criteria were: (1) MS clinical relapse or treatment with corticosteroid therapy within 6-months prior to examination, (2) patients experiencing major depression or cognitive decline, (3) orthopedic disorders that could negatively affect balance, (4) pregnancy, (5) blurred vision, or (6) cardiovascular disorders. All participants gave informed consent prior to participation. Additionally, written informed consent for publication of clinical images was obtained from the participants. Approval was obtained from the Sheba Medical Center Independent Ethics Committee prior to commencement of the study.

### Sample size

The sample size estimate was based on extrapolations from our preliminary data and other related studies examining the effects of VR on balance capabilities in PwMS. Accordingly, we used the effect size (0.9) of the functional reach test (FRT) for calculations. Power was set at 80 %, alpha was set at 5 % and we accounted for dropout rate of 10 %. Using a relatively conservative estimate, a total of 32 subjects (16 in each group) would be required to detect differences between the two treatment groups assuming non-inferiority with moderate correlations among covariates (R-squared = 0.50).

### Study design

After consenting to participate and fulfilling the inclusion criteria, thirty-two people with relapsing-remitting MS were equally randomized into the VR intervention group or the conventional exercise program control group with a 1:1 allocation ratio. For randomization, sealed envelopes were prepared in advance and marked on the inside with an O or X. Randomization was performed one hour prior to the start of the pretest by a physical therapist who was not involved in the assessment or treatment of the subjects. The intervention period of both groups was identical, 6 consecutive weeks, two sessions per week, 30 min sessions.

Outcome measures were collected twice, upon initiation of the intervention programs and at termination of the 6-week intervention period. All measurements were completed by an experienced physical therapist specialized in neurological rehabilitation, blinded to participant grouping. A research coordinator documented all training and examination sessions. Within the first three weeks of the study period, one subject from each group withdrew from the training program due to difficulties in reaching the MS Center. Thus, data from 30 patients (19 females, 11 males; mean age, (S.D.) = 45.2 (11.6) years; mean EDSS (S.D.) = 4.1 (1.3), mean disease duration (S.D.) = 11.0 (8.9) years), were analyzed.

### The VR intervention program

**Fig. 1:**
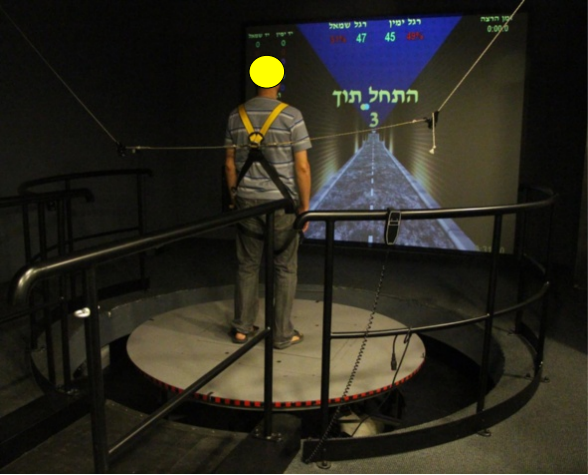
The CAREN Integrated Virtual Reality System

**Fig. 2:**
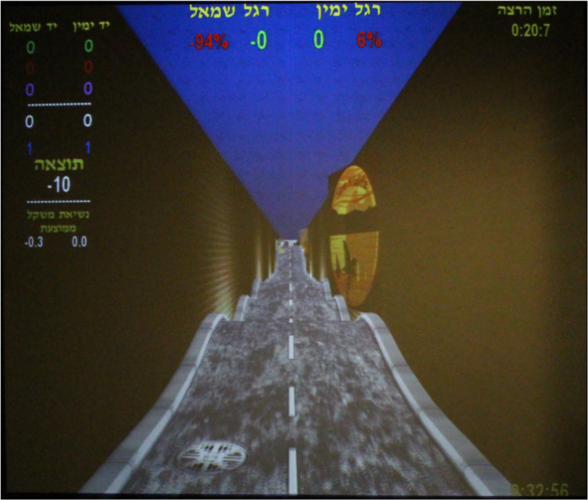
The virtual road scene projected on the system’s screen

The VR system used was the CAREN Integrated Reality System with D-flow software. This system, designed by Motek Medical BV, works in real-time, enabling the creation of a variety of controlled and repeated simulated environments via dedicated software which includes 3D visual, sound, and proprioceptive stimuli. The following components are incorporated in the system:

1. Motion Platform - CAREN consists of an electro-hydraulic 2 m diameter motion platform (Rexroth Hydraudyne, MOTEK, Micro motion) that can be manipulated by 6° of freedom (x-y-z translation and pitch-roll-yaw rotation). The movement of the platform is either driven by the subject’s movements or preprogrammed in synchrony with function curves that define a specific pathway in the virtual environment (Fig. 1).
2. Projection: The virtual scene is projected on a large screen (3 m × 2.5 m). In the present study, a VR setting entitled ‘road scene’ was used which requires subjects to stand on a motion platform while maintaining their balance and advancing along a pre-defined road. The scene, consisting of a virtual road, bounded on both sides by walls is projected onto the screen, in front of the subject. The road itself has flat, straight and vertical bumpy sections (movement along the y-axis), right and left tilts (rotation of ‘z axis’ - “roll”) and right and left translations (movement along the ‘x axis’ - “sway”). The platform’s movement is correlated with the visual stimulus (i.e., when the subject arrives at a bump on the screen, the platform elevates. When the road tilts, the platform tilts accordingly in the same direction). The length of the road is 1230 m. The road’s default velocity was set at 30 m/min which is equal to 1.8 km/h (Fig. 2).

The instructions during VR training were as follows: “In front of you will appear a road that advances toward you. Your goal is to keep your balance in response to changes in the slope or direction of the road”.

**Fig. 3:**
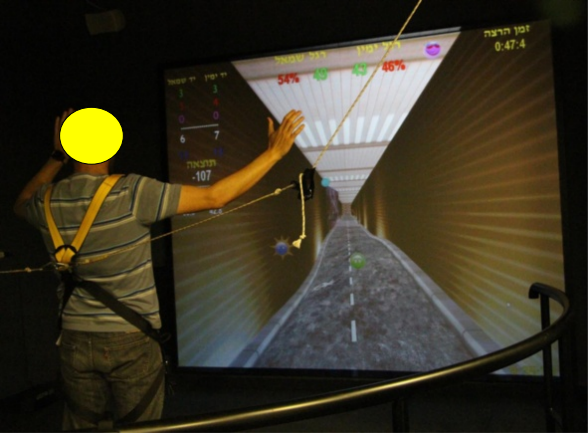
The virtual road scene while intercepting moving targets

The VR training session included a secondary task: intercepting 18 moving targets (a colored ball of 12.5 cm diameter) appearing above the road. Each target can be intercepted when appearing no further than 20 cm from the subject’s hands (which are reflected on the screen as a 4.5 cm in diameter ball). The targets appear one at a time, at pre-set points along the road, alternately on the right and left sides of the subject’s body. Each ball is displayed for 5 s and then disappears (unless intercepted). In the event of this occurring, the participant is instructed to maintain his balance (as before) but also to try and reach out with his arms to touch the virtual balls (Fig. 3). After intercepting a ball, the subject is instructed to return to the ‘home position’ (hands at the sides of the body).

In either scene, participants and/or the physical therapist were allowed to increase the system’s setting (platform’s tilt, amplitude and velocity (range 3.0-1.0 km/h)) once they felt that the balance challenge was too low, thus the VR training settings for each participant varied slightly in order to maximize the effectiveness of balance training. During all training sessions, participants wore a safety harness that did not support any body weight. Additionally, a physical therapist stood behind the patient protecting him from falling.

Patients participated in 12 sessions of CAREN VR over six weeks, with a total of 30 min of balance training during each session. Rest breaks were allowed if requested, but were not included in the overall practice time. Typically, the participants took three rest breaks during each practice session, each lasting approximately 3–4 min. The training session lasted 45–50 min. Participants were instructed to continue their regular physical activities between intervention sessions.

To date, there is no consensus as to the optimal duration, frequency and intensity of balance training intervention programs in MS. According to Gunn et al’s, systematic review, the duration of balance programs in PwMS vary between 3 and 12 weeks, while intense practice sessions per week vary between 30 and 210 min [11]. Therefore, the intervention timeframe was based on our desire to remain effective in terms of balance training frequency, nonetheless, minimize the difficulties involved in transporting the patients to the MS Center, as this could have probably increased the study’s dropout rate.

### The conventional exercise program

In each of the 12 sessions, the participants underwent 10 min of stretching exercises and 20 min of intervention. The training protocol included a combination of static postural control, weight shifting and perturbations exercises. During the static postural control exercises, the patients were encouraged to stand motionless on pieces of unstable foam with eyes open or closed for approximately one minute (depending upon the patient’s ability). The difficulty was increased by adding more unstable pieces and reducing the base of support. Weight shifting was achieved by a physical therapist who threw a ball in numerous directions. The patients had to catch the ball approximately 30 times by reaching and stepping. The difficulty was increased by changing the ball size, throwing further distances and at faster speeds. As to the perturbations exercises, patients were encouraged to stand on an unstable base (i.e. a wobble board) while the physical therapist deliberately pushed the top of the board in a downward direction, on different places and at various speeds. During all sessions, the patient was supervised by the physical therapist in order to prevent falls. The conventional balance training protocol was similar to the protocol used by Yen et al. in their study of people with Parkinson’s disease [28].

### Instrumented posturography

Posturography parameters were obtained from the Zebris FDM-T Treadmill data (Zebris® Medical GmbH, Germany) taken at the Center of Advanced Technologies in Rehabilitation, Sheba Medical Center, Israel. A description of the Zebris treadmill is detailed in our previous report on postural control, falls and fear of falling in PwMS [7].

A set of outcome measures taken from the center of pressure (CoP) trajectories during the static stance were:

1. Ellipse sway area (mm2): defined as the 95 % confidence ellipse for the mean of the CoP anterior, posterior, medial and lateral coordinates.
2. CoP path length (mm): defined as the absolute length of the CoP path movements throughout the testing period.
3. Sway rate (mm/s): defined as the mean speed of movement of the CoP during the testing period.
4. The average pressure distribution of the left and right feet expressed in bodyweight (%). Additionally, the bilateral pressure distribution asymmetry score was calculated as the absolute difference in pressure distribution between the legs. In a perfect symmetrical stance, this variable is zero.

Each subject completed a sequence of three consecutive tests under two different task conditions (eyes open and eyes closed) with a 1-min break between tasks. Each task was repeated three times, for 30 s, followed by a 30 s rest period. Posturography results are presented as the mean value of the three tests. The CoP path length was selected as the primary outcome in view of the fact that previous investigations have confirmed a significant association between CoP path length to falls and white matter tract damage in PwMS [29].

### Clinical balance tests

The FRT assesses the subject’s stability by measuring the maximum distance an individual can reach forward while standing in a fixed position. A longer reaching distance indicates improved postural control [30].

The Berg Balance Test (BBS) consists of a set of 14 simple balance related tasks, ranging from rising from a sitting position to standing on one foot. The score ranges from 0 to 56. A score of 41–56 indicates a low fall risk, 21–40 = medium fall risk and 0 –20 = high fall risk [31].

The Four Square Step Test (FSST) is a timed test, intended to challenge the rapid change in direction while stepping forward, backward and sideways over a low obstacle. The faster the time measured to perform the task, signifies a superior level of dynamic balance abilities [32]. The minimal detectable change estimate for the FSST in MS is 4.6 s [33].

The Falls Efficacy Scale International (FES-I) is a patient self-reported questionnaire used to assess the level of concern relating to falls during 16 activities of daily living, ranging from basic to more demanding activities, including social activities that may contribute to quality of life. The scores can range from 16 to 64; the higher the score, the more the fear of falling [34].

### Statistical analysis

Data analysis was performed using IBM SPSS statistics software (Version 22.0 for Windows, SPSS Inc. NY, USA). Data was initially examined for normality violations, outliers, errors and missing values. Groups were compared at baseline using the *t*-test for independent samples for continuous variables and the chi-square test for categorical data. All outcome variables showed normal distribution. The repeated measure analysis of variance (ANOVAs) was selected to examine the effects of each group and determine whether a specific intervention had any advantage in terms of primary and secondary outcome balance measures. A *P*-value in each case < .05 was considered significant.

## Results

**Fig. 4:**
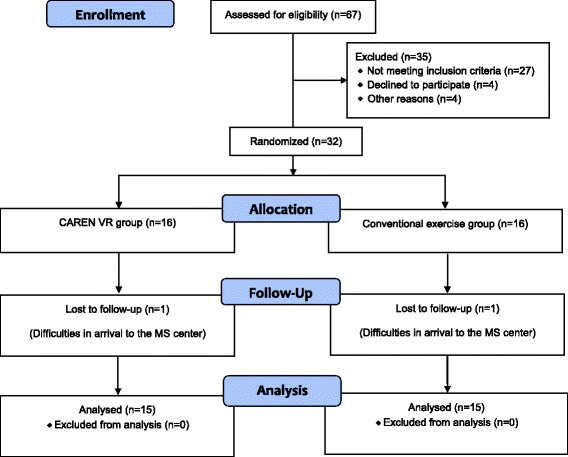
A flow chart of the study

A flow chart of the study is shown in Fig. 4. Demographic and clinical data of the 30 subjects who fulfilled the study is presented in Table 1. All participants participated in at least 10 (out of the planned 12) training sessions. No significant differences in terms of baseline values were observed between the VR and control group. No adverse or harmful events were reported in both groups. Table 2 shows the values of all outcome measures. Both groups showed a main effect of time on the CoP path length with eyes open (F = 5.278, *P* = .024), sway rate with eyes open (F = 5.852, *P* = .035), FRT (F = 20.841, *P* = .001), FSST (F = 9.011, *P* = .031) and the FES-I self-reported questionnaire (F = 17.815, *P* = .023). In addition, significant differences supporting VR intervention were observed for the group x time interactions of the FRT (F = 10.173, *P* = .009) and FES-I (F = 6.710, *P* = .021). There was no main effect of time in both groups in terms of posturography parameters performed in the closed eyes condition.

**Table 1:** Demographics and clinical characteristics of the study group

Variable	Mean (S.D.)		*P*- Value
	VR (*n* = 15)	Control (*n* = 15)	
Age (years)	47.3 (9.6)	43.9 (10.6)	0.433
Gender			
Male	5	6	---
Female	10	9	---
Disease duration (years)	11.6 (7.7)	10.4 (6.5)	0.613
Height (cm)	167.4 (7.1)	168.1 (8.8)	0.459
Body mass (kg)	72.8 (11.4)	70.8 (12.5)	0.482
EDSS	4.5 (1.6)	3.9 (1.3)	0.327
Pyramidal	2.7 (0.9)	2.5 (0.8)	0.226
Cerebellar	1.8 (1.0)	1.7 (0.8)	0.241
Sensory	1.4 (1.0)	1.2 (1.0)	0.262

*EDSS* Expanded disability status scale

**Table 2:** Comparison of the study outcome measures

Parameters	VR group (*n* = 15)			Standardized exercise group (*n* = 15)			*F* (*P*-value) for time factor	*F* (*P*-value) for group X time factor
	Pre-intervention	Post-intervention	Mean difference	Pre-intervention	Post-intervention	Mean difference		
Posturography- eyes open								
CoP path length (mm)	345.3 (76.2)	290.5 (105.3)	−54.8 (52.4)	313.5 (89.5)	272.9 (78.4)	−40.6 (32.9)	5.278 (0.024)*	1.569 (0.226)
Sway rate (mm/s)	17.0 (6.3)	13.0 (6.5)	−3.0 (3.4)	16.1 (6.3)	12.6 (4.4)	−3.5 (3.4)	5.852 (0.035)*	0.997 (0.232)
Ellipse sway area (mm^2^)	309.0 (116.3)	319.8 (130.5)	10.8 (74.2)	309.0 (116.3)	319.8 (130.5)	10.8 (74.2)	0.754 (0.872)	0.659 (0.624)
Pressure distribution difference (%)	9.4 (7.4)	9.5 (6.3)	0.1 (6.1)	9.4 (7.4)	9.5 (6.3)	0.1 (6.1)	0.076 (0.984)	0.085 (0.892)
Posturography- eyes closed								
CoP path length (mm)	392.3 (112.3)	333.1 (156.4)	−59.2 (98.0)	376.1 (102.9)	343.2 (146.9)	32.9 (102.4)	1.023 (0.189)	0.067 (0.348)
Sway rate (mm/s)	20.1 (15.3)	18.4 (9.8)	−1.7 (7.7)	22.4 (12.4)	18.9 (11.8)	−3.5 (8.0)	0.921 (0.239)	0.910 (0.296)
Ellipse sway area (mm^2^)	378.3 (109.5)	342.5 (100.5)	−35.8 (94.2)	363.3 (99.1)	338.8 (109.4)	−24.5 (72.1)	1.351 (0.137)	0.004 (0.981)
Pressure distribution difference	9.9 (7.9)	10.3 (8.3)	0.4 (5.1)	9.6 (7.1)	10.7 (6.3)	1.1 (5.9)	0.045 (0.887)	0.019 (0.675)
Clinical balance tests								
FRT (cm)	30.1 (5.0)	34.8 (6.9)	4.8 (4.1)	27.6 (6.4)	30.2 (4.9)	2.6 (3.2)	20.481 (0.001)*	10.173 (0.009)*
BBT	46.8 (9.6)	47.9 (6.4)	1.1 (4.2)	43.3 (7.1)	44.6 (4.9)	1.3 (5.2)	1.541 (0.215)	1.794 (0.561)
FSST (s)	16.2 (7.0)	12.7 (6.4)	−4.5 (5.0)	14.2 (7.1)	11.7 (5.9)	−3.5 (6.1)	9.011 (0.031)*	1.250 (0.361)
FES-I questionnaire	36.4 (9.7)	29.4 (7.8)	−7.0 (4.3)	32.9 (10.3)	28.6 (5.8)	−4.3 (6.3)	17.815 (0.023)*	6.710 (0.021)*

*CoP* Center of pressure, *FRT* Functional reach test, *BBT* Berg balance test, *FSST* Four step square test, *FES*-*I* Falls Efficacy Scale International

Values are expressed as mean (S.D). *P-Value <0.05

## Discussion

The primary objective of the current study was to investigate the effect of VR training on balance in PwMS, utilizing the CAREN system. To our knowledge, our study is the first RCT conducted with the CAREN system in the neurological population. Following six weeks of VR training, both MS groups demonstrated a significant effect on clinical balance tests and 2 (out of 8) posturography measures. Additionally significant differences for the group x time interactions, in favor of the VR group were demonstrated in the functional reach test and the fear of falling questionnaire.

Generally, our results resemble several trials performed on other neurological populations such as stroke survivors, people with Parkinson’s disease, and elderly fallers [15–17]. In a recent Cochrane systematic review (37 trials, *n* = 1019) examining the efficacy of VR training on stroke rehabilitation, the authors reported that VR was beneficial in improving the activities of daily living (ADL) function [35]. However, the same group noted that there was insufficient evidence as to the effect of VR on gait speed or global motor function. At present it is still undetermined as to which VR characteristics are most important for gait and balance training and whether these effects are sustained over an extended period of time.

Worth noting, due to the wide variety of VR rehabilitation systems, comparisons between studies are confusing and could be misleading. This refers mainly to systematic reviews that inadvertently defined low-cost commercial exergaming devices (e.g Nintendo Wii, Sony eye-toy, Microsoft Kinect) as VR training tools.

We note several significant differences between these two training tools. Originally, exergaming devices were developed for playing needs of healthy children. Therefore, it is questionable whether the playing scenarios offered by these devices are relevant for the rehabilitation needs of patients with mobility difficulties. According to a recent systematic review, the addition of Wii gaming to conventional rehabilitation in patients with chronic stroke, significantly improved in performance in the Timed up and go test but not in the other physical measures (e.g functional independence measure score, BBS and anterior-posterior sway). Furthermore, the pooled effect statistic was small and not beyond the minimal detectable change [36]. Moreover, commercial exergaming devices do not allow scenario adjustments that in many cases are necessary to meet the physical abilities and treatment goals of the patient. In contrast, VR system training scenarios can be set up according to the patient’s abilities and progress during the intervention program. Additionally, VR training can be conducted in a controlled environment in order to regulate mechanical and visual cues. As such, rehabilitation outcomes may be specifically investigated without the risk of confounding variables.

In line with the distinction between commercial exergaming and VR training, our study explored a medically-oriented VR system, the CAREN system. Previous studies examining the efficacy of this device on balance performance primarily studied patients with orthopedic and vestibular pathologies, with promising results [24, 25, 37]. The CAREN system challenges subjects both physically and cognitively in realistic, interactive and controlled environments. Patients can interact with the system due to the integrated force plates measuring weight shifting and body motion. Detailed visual displays are projected onto a large screen and move in synchronization with the platform and the subjects’ movements. Engagement is further enhanced with realistic sounds and scents. Additionally, treatment on the CAREN can allow the subject to identify problematic balance situations that occasionally occur in the community but cannot be replicated in traditional physical therapy.

To date, this is the first RCT study examining the CAREN system in the MS population. In accordance with our findings, we are confident that this system is safe and feasible for use in balance rehabilitation programs for PwMS. Notably, none of the patients reported any motion sickness during the practice sessions. In addition, although information regarding engagement and motivation while practicing with the CAREN system wasn’t systematically collected, we report that participants in the VR group barely missed a practice session. Moreover, although a similar appearance rate was recorded in the conventional exercise program, at termination, most VR patients indicated an interest in continuing with the VR system.

We hypothesize that this unofficial outcome is correlated with the fear of falling self-report questionnaire findings. Participants in the VR group significantly reported less fear of falling compared to the control group. We speculate that the CAREN’s unique environment is related to this outcome, as has been raised in Collins et al’s recent systematic review [37]. While most MS patients are familiar and have a basic knowledge regarding physical therapy sessions, this is not the case with interventions based on high technology VR systems. As noted previously, the CAREN system includes a huge screen, surround audio speakers and a large platform tilting and rolling in all directions. Moreover, this sophisticated system is placed in a dedicated darkened room and activated by a technician. There is a possibility that all these factors, combined with the new experience, had a positive effect on the patient’s level of confidence. Nevertheless, future studies are needed to confirm this assumption. In line with this finding, Duque et al. [15] examined the effectiveness of a 6-week balance training program in 60 community older subjects using a VR system. The authors reported a significant reduction in the incidence of falls and fear of falling at termination of the intervention period in the VR group compared with the control group [15].

Our study has some strengths and limitations. The major strength of this study is that this is the first demonstration of the beneficial effect of balance training on balance parameters and fear of falling in PwMS by using a novel, accurate, safe and effective VR method.

Another contribution of the study was the use of computerized posturography to document changes in balance parameters. One of the advantages of this method is a more precise determination of changes in balance parameters, compared to the usual clinical balance tests [38]. This data could be valuable for comparison in future studies examining postural control in PwMS.

### Limitations

Performing a double-blind, controlled trial to test this system is clearly unfeasible; therefore we decided to use an open approach in which both the participants and medical team were aware of the interventions. However, to prevent any assessment bias, different physiotherapists with no access to the subjects’ data were specifically assigned to perform either assessment or training. Another limitation of the study was the absence of a follow up examination; consequently, we were unable to report whether the improvements demonstrated by the VR participants were maintained over time. This important information deserves further exploration in future longitudinal clinical trials. Finally, in light of the fact that this is a pilot study, the sample size was relatively small. Nevertheless, our results may serve future studies to generate effect sizes, which can be used to power a larger clinical trial aimed at improving postural control, reducing falls in PwMS and other neurological populations based on VR technology.

## Conclusions

We have successfully demonstrated that balance training based on the CAREN device is an effective method in balance training for PwMS. Although it is not intended to replace, but rather complement other balance intervention programs, VR training offers a safe and well-accepted intervention with appropriate levels of effectiveness and adherence. While traditional therapy methods will continue to be employed in the clinic, VR based balance training programs have demonstrated the ability to directly influence balance performance. Nevertheless, it is worth noting that while the CAREN system has numerous clinical advantages, the financial and spatial requirements for the system may preclude this device from being a rehabilitation aid in several medical centers and in cases of MS patients treated in the community and/or restricted to their homes.

## Abbreviations

ANOVA: Analysis of variance
BBS: Berg balance scale
CAREN: Computer assisted rehabilitation environment
CoP: Center of pressure
EDSS: Expanded Disability Status Scale
FES-I: Falls Efficacy Scale International
FRT: Functional reach test
FSST: Four square step test
MS: Multiple sclerosis
PwMS: Persons with MS
VR: Virtual reality
